# Evaluation of Real-World Tumor Response Derived From Electronic Health Record Data Sources: A Feasibility Analysis in Patients With Metastatic Non–Small Cell Lung Cancer Treated With Chemotherapy

**DOI:** 10.1200/CCI.24.00091

**Published:** 2024-08-15

**Authors:** Brittany A. McKelvey, Elizabeth Garrett-Mayer, Donna R. Rivera, Amy Alabaster, Hillary S. Andrews, Elizabeth G. Bond, Thomas D. Brown, Amanda Bruno, Lauren Damato, Janet L. Espirito, Laura L. Fernandes, Eric Hansen, Paul Kluetz, Xinran Ma, Andrea McCracken, Pallavi S. Mishra-Kalyani, Yanina Natanzon, Danielle Potter, Nicholas J. Robert, Lawrence Schwartz, Regina Schwind, Connor Sweetnam, Joseph Wagner, Mark D. Stewart, Jeff D. Allen

**Affiliations:** ^1^ Friends of Cancer Research, Washington, DC; ^2^ American Society of Clinical Oncology, Alexandria, VA; ^3^ Oncology Center of Excellence, US FDA, Silver Spring, MD; ^4^ ConcertAI, Cambridge, MA; ^5^ Guardian Research Network, Spartanburg, SC; ^6^ Syapse, San Francisco, CA; ^7^ Syneos Health, Morrisville, NC; ^8^ Flatiron Health, New York, NY; ^9^ Ontada, Boston, MA; ^10^ COTA, Inc, New York, NY; ^11^ US FDA, Silver Spring, MD; ^12^ IQVIA, Durham, NC; ^13^ Memorial Sloan Kettering Cancer Center, New York, NY; ^14^ Tempus AI, Inc, Chicago, IL

## Abstract

**PURPOSE:**

Real-world data (RWD) holds promise for ascribing a real-world (rw) outcome to a drug intervention; however, ascertaining rw-response to treatment from RWD can be challenging. Friends of Cancer Research formed a collaboration to assess available data attributes related to rw-response across RWD sources to inform methods for capturing, defining, and evaluating rw-response.

**MATERIALS AND METHODS:**

This retrospective noninterventional (observational) study included seven electronic health record data companies (data providers) providing summary-level deidentified data from 200 patients diagnosed with metastatic non–small cell lung cancer (mNSCLC) and treated with first-line platinum doublet chemotherapy following a common protocol. Data providers reviewed the availability and frequency of data components to assess rw-response (ie, images, radiology imaging reports, and clinician response assessments). A common protocol was used to assess and report rw-response end points, including rw-response rate (rwRR), rw-duration of response (rwDOR), and the association of rw-response with rw-overall survival (rwOS), rw-time to treatment discontinuation (rwTTD), and rw-time to next treatment (rwTTNT).

**RESULTS:**

The availability and timing of clinician assessments was relatively consistent across data sets in contrast to images and image reports. Real-world response was analyzed using clinician response assessments (median proportion of patients evaluable, 77.5%), which had the highest consistency in the timing of assessments. Relative consistency was observed across data sets for rwRR (median 46.5%), as well as the median and directionality of rwOS, rwTTD, and rwTTNT. There was variability in rwDOR across data sets.

**CONCLUSION:**

This collaborative effort demonstrated the feasibility of aligning disparate data sources to evaluate rw-response end points using clinician-documented responses in patients with mNSCLC. Heterogeneity exists in the availability of data components to assess response and related rw-end points, and further work is needed to inform drug effectiveness evaluation within RWD sources.

## Teaser

Consistency of real-world response taken from EHR data in patients with mNSCLC treated with chemotherapy.

## INTRODUCTION

Despite the rigor of clinical trials, further understanding of a therapy's effectiveness may still be needed. The use of real-world data (RWD) to generate real-world evidence (RWE) may fill these gaps and support evaluation of therapeutic effectiveness. RWD may more readily capture the heterogeneity of the intended use population, provide information on long-term safety and effectiveness, and identify off-label use.^1^ Recent efforts to increase research on and support use of RWE include the 21st Century Cures Act,^2^ Prescription Drug User Fee Act VI^3^-VII,^4^ the Food and Drug Omnibus Reform Act of 2022,^5^ and President Biden's Cancer Moonshot.^6^ To support drug development and regulatory decision making, there is a need to align on and further evaluate the use of RWD, including standardizing data elements, aligning definitions, and reproducing methodology across real-world (rw) data sets.

**CONTEXT**

**Key Objective**
To develop an aligned methodology for assessing real-world response to treatment across disparate data sources.
**Knowledge Generated**
This methodological exercise supports the ability to align disparate data sources to evaluate rw-response in an aligned patient population. Real-world response end points using clinician-documented response show relative consistency across data sources.
**Relevance**
Using real-world data (RWD) in clinical practice can greatly enhance the understanding of treatment effectiveness, inform personalized care plans, and identify emerging trends in patient populations, ultimately improving health care quality and outcomes. This study evaluated patients with metastatic non–small cell lung cancer (mNSCLC) who were treated with first-line platinum doublet chemotherapy. It focused on the consistency and availability of data components in RWD sources, such as clinician assessments and radiology reports. The objective was to develop a methodology for determining real-world response (rw-response) and to explore its potential application in oncology research. The study demonstrated the feasibility of integrating diverse data sources to evaluate rw-response end points using clinician-documented responses in patients with mNSCLC. It highlighted the relative consistency of real-world response, underscoring the potential of RWD to support oncology research and inform clinical decision making.

Friends of Cancer Research (*Friends*) previously convened key stakeholders to participate in collaborative pilots^7-9^ to define rw-end points, including rw-overall survival (rwOS), rw-time to treatment discontinuation (rwTTD), and rw-time to next treatment (rwTTNT), and align these definitions across multiple RWD sources to enhance generation of RWE on patient outcomes. These pilots highlighted areas of concordance in the direction and magnitude of treatment effect measured through rw-end points across data sources when using a common research protocol. However, the projects found the common limitation that progression events were not consistently captured, requiring an additional concerted effort to evaluate approaches for capturing end points assessing change in tumor burden, such as objective response rate (ORR) and progression-free survival (PFS).

ORR is an informative regulatory measure that can be used as an end point in single-arm trials, as causality is reasonably inferred (ie, tumors do not typically shrink spontaneously). Response rate is also evaluated earlier in the treatment course and may be reasonably likely to predict clinical benefit (ie, PFS and OS).^10^ The duration and magnitude of response is important to understand the treatment-response trajectory and to ascribe clinical meaningfulness. Within clinical trials, RECIST 1.1 outlines a standardized approach (ie, consistent and objective mode of evaluation and cadence of assessment) to capture the response of solid tumors to an oncology treatment. However, there are challenges with characterizing rw-response in solid tumors, as the components necessary to measure RECIST-based response are not often accessible or available in the electronic health record (EHR) or assessed in a standardized manner outside of a protocol-driven study. Recognizing the increased heterogeneity of routine clinical practice, when compared with clinical trials, this pilot project sought to (1) understand the availability and feasibility of using specific RWD elements to assess rw-response, (2) evaluate the potential to ascertain rw-response using available data elements from the EHR, and (3) evaluate the consistency of these measures across data sources.

## MATERIALS AND METHODS

### Standardization of Methods

A collaborative partnership of RWD providers, pharmaceutical companies, academics, and government agencies jointly developed the common protocol and statistical analysis plan, including definitions on patient selection criteria, data elements, and outcomes (Data Supplement, Tables S1-S8). Each RWD provider (cohort) assessed their deidentified, patient-level EHR data to report uniform summary results (Data Supplement). Contributing data providers included ConcertAI, COTA Inc, Flatiron Health, Guardian Research Network and IQVIA, Ontada, Syapse, and Tempus AI.

### RWD Cohort Development

**FIG 1.**
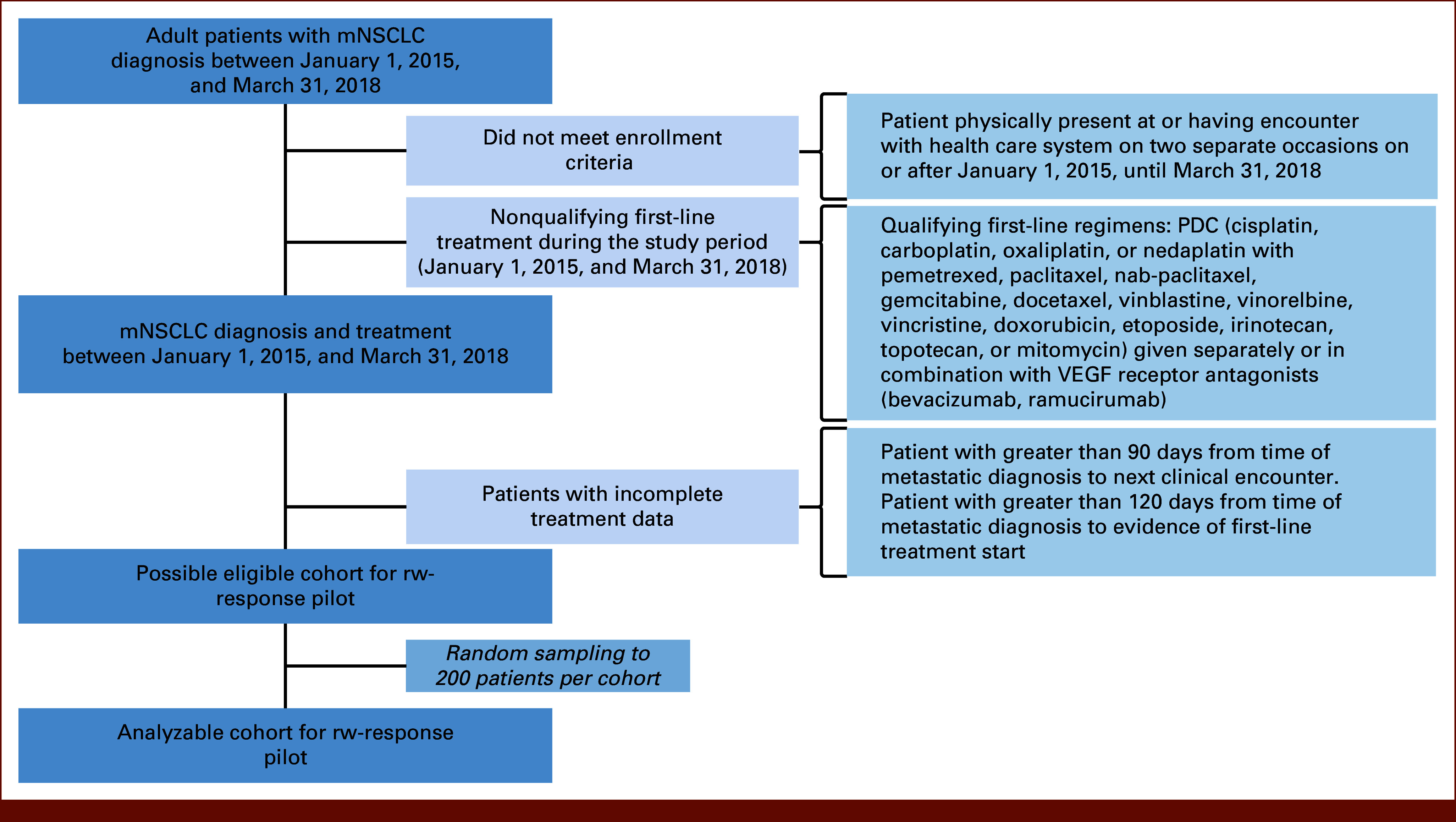
Flow diagram. mNSCLC, metastatic non–small cell lung cancer; PDC, platinum doublet chemotherapy; VEGF, vascular endothelial growth factor.

Each cohort identified adult patients (age 18 years or older at metastatic diagnosis) with histologically confirmed metastatic non–small cell lung cancer (mNSCLC) by structured or abstracted data, diagnosed between January 1, 2015, and March 31, 2018 (inclusive) in their databases, a time frame reflective of cohorts selected for previous pilots.^7-9^ All cohorts received institutional review board approval or exemption. Patients received first-line (1L) treatment with platinum doublet chemotherapy (PDC) regimens with or without vascular endothelial growth factor (VEGF) receptor antagonists (Data Supplement, Fig 1). Eligible patients were documented as physically present at a practice or having an encounter in the database on at least two separate occasions, and patients were excluded if there was incomplete treatment data (Data Supplement). Of the eligible patients, each data provider performed random sampling to achieve a cohort size of 200 patients. After sampling, an additional 20 patients were excluded from cohort G for not meeting eligibility criteria. This sample size was chosen to ensure uniformity across cohorts and for feasibility reasons, because of the level of data curation necessary. Clinical and demographic characteristics were summarized using descriptive statistics.

### Assessment of Availability of Response Data Components

Cohorts assessed the availability of core data components during the assessment period. Components included images (magnetic resonance imaging [MRI], positron emission tomography-computed tomography [PET-CT], CT, and other), image reports (MRI, PET-CT, CT, and other), and clinician assessment of response (as stated in notes, where response evidence was referenced from imaging, symptoms, laboratory results, physical examination, pathology reports, other sources, or was not specified). The data component assessment was divided into two periods, baseline (time from the metastatic diagnosis date to the day before the start of 1L therapy, defined as the index date) and postbaseline (time from the index date up to the earliest of the start of new [second-line] treatment, 30 days after the last administration of 1L treatment, death, or data cutoff), to identify both baseline and postbaseline images or image reports for response assessment. Evaluation of clinician assessment of response was only conducted in the postbaseline period. Results were summarized for the proportion of patients in each cohort with each data component available within the assessment period. Medians and IQRs were reported for the number and timing of data components per patient. The component source (image modality and indication for image reports, or source for clinician response assessment in the record) was treated as a categorical variable and reported as a proportion of the total number of available data components. Additional statistical considerations are described in more detail in the Data Supplement.

### Methodology for rw-Response End Points and Parameter Estimation

Clinician assessment of response was used to determine rw-response for all patients using the categories rw-complete response (rwCR), rw-partial response (rwPR), rw-stable disease (rwSD), rw-progressive disease (rwPD), rw-mixed response (rwMR), and not evaluable (NE; Data Supplement, Table S3). The rw-best overall response (rwBOR) was defined as the patient's best response, where rwCR was the most favorable, followed by rwPR, rwSD, rwPD, rwMR, and NE. The rw-response rate (rwRR) was defined as the proportion of patients with a rwBOR of rwCR or rwPR among all patients, including patients with no assessment. Patients were further classified as responders (patients with at least one response assessment of rwCR or rwPR), nonresponders (patients with at least one response assessment, but none that were rwCR or rwPR), or no response data (patients with no clinician assessment of response). Patient follow-up time and demographic and clinical characteristics were analyzed by this three-category response classification. The rw-duration of response (rwDOR) was defined as the length of time from the date of the first documented assessment of rwCR or rwPR after the index date to the date of the first subsequent documented assessment of rwPD, rwMR, or death. For patients without progression events, rwMR, or death, the patient was censored at their last known response assessment of rwCR, rwPR, or rwSD, or the date of treatment discontinuation, whichever came first. Median rwDOR was estimated using Kaplan-Meier methods, reporting 3-, 6-, and 9-month estimates with 95% CIs. Because of the variability in response assessment frequency, an approach accounting for interval censoring was included for the estimation of rwDOR, which was calculated and described using a nonparametric estimation on the basis of Turnbull's algorithm, reporting median, 3-, 6-, and 9-month estimates with 95% CIs. Sensitivity analyses were also conducted to assess the influence of the different proportions of patients with certain prognostic factors of response or potential confounding factors. Real-world RR and rwDOR were assessed in the following subgroups: patients not receiving other treatment modalities during the 1L therapy (eg, surgical resection and radiation), patients not receiving other allowed agents (VEGF antagonists) during the 1L therapy, and patients without brain and/or bone only metastases.

### Association Between rw-Response and Time to Event rw-End Points

The rw-end points of rwOS, rwTTD, and rwTTNT were measured as previously defined^8^ (Data Supplement, Tables S4 and S5). The rw-end points were described by medians with 95% CIs for all patients for each of the cohorts in the data sources. Subgroup analyses were conducted, calculating rwOS, rwTTD, and rwTTNT for responders, nonresponders, and no response data subgroups. Data by subgroup were summarized using Kaplan-Meier curves and described by medians and 95% CIs.

## RESULTS

### Cohort Selection and Characteristics

**FIG 2.**
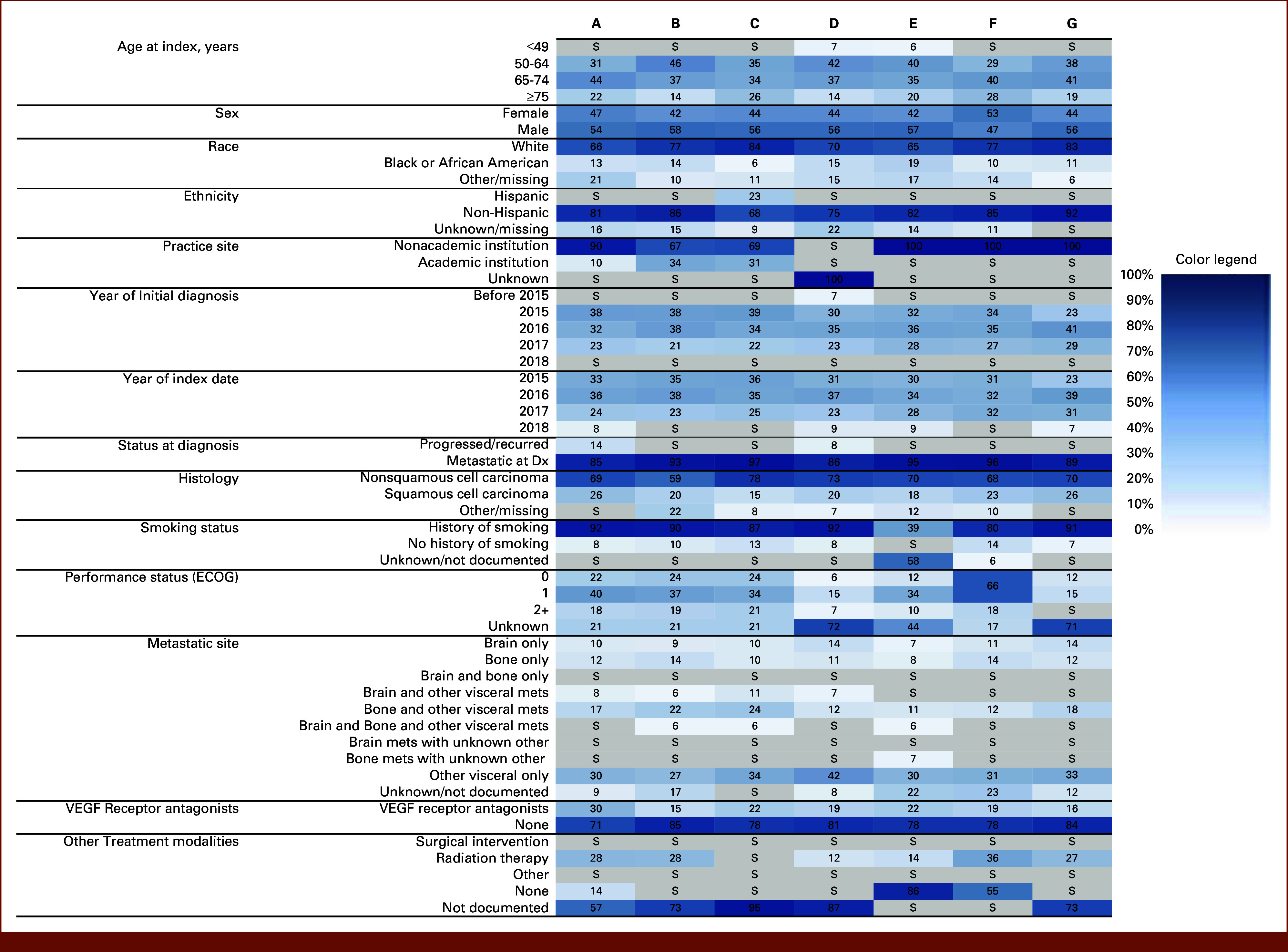
Demographic and clinical characteristics of cohorts. Numbers represent the proportion of patients in each category. Shading denotes the proportion of patients from white (0%) to dark blue (100%) to aid in visual comparison across cohorts. Data are suppressed (S, in gray) if ≤5%. Dx, diagnosis; ECOG, Eastern Cooperative Oncology Group; VEGF, vascular endothelial growth factor.

After applying all eligibility criteria, the most common reason for patient exclusion was receipt of a nonqualifying 1L regimen (eg, immunotherapy) during the assessment period (Data Supplement, Table S9). Most cohorts had patients with complete historical treatment data. Demographic characteristics were similar across cohorts including largely White and non-Hispanic patients, with some missingness of demographic variables noted. Variability was observed in type of practice site (Fig 2), with most patients from nonacademic institutions. Most patients had metastatic disease at initial diagnosis, with nonsquamous histology and a history of smoking. Most patients were not treated with VEGF receptor antagonists or other treatment modalities (eg, concomitant surgery or radiation). Eastern Cooperative Oncology Group status was not reported consistently, and variability was observed for documentation of sites of metastases.

### Availability of Core Data Components for Evaluating rw-Response

**FIG 3.**
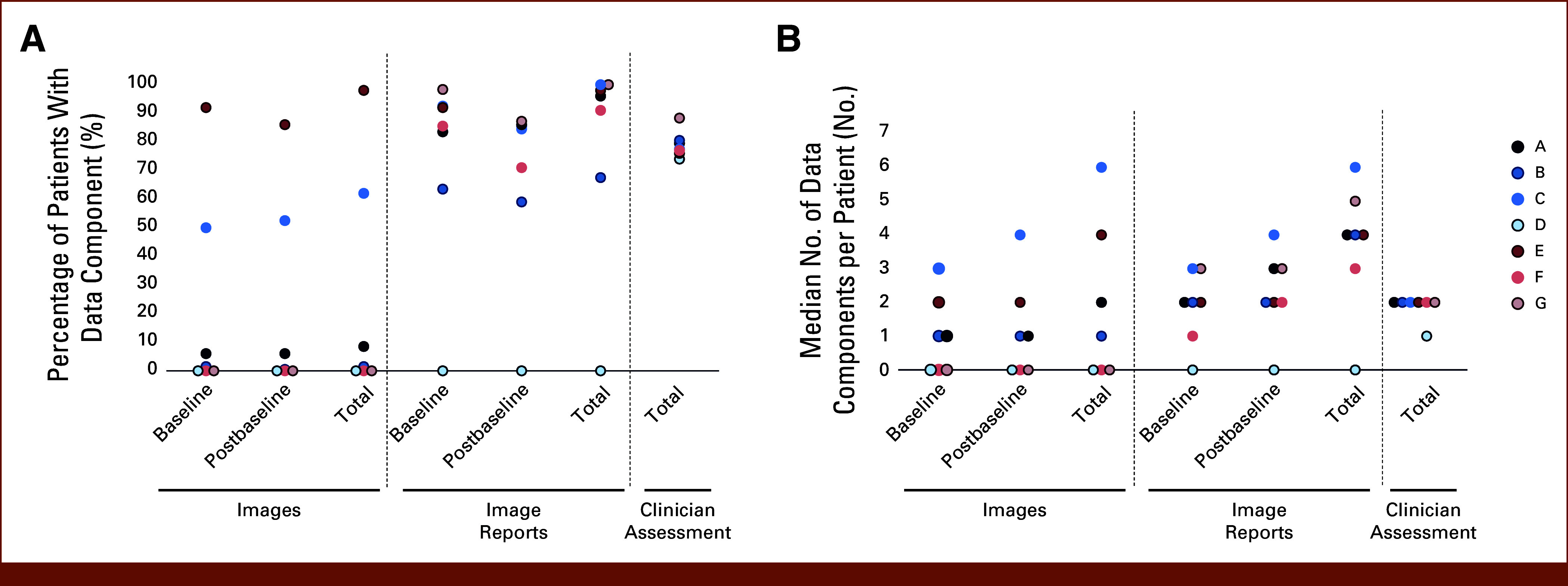
Availability of rw-response assessment data components. Dot plots depict (A) the percentage of patients in each cohort with each data component and (B) median number of data components per patient. The median number of data components is calculated only for patients with at least one data component in the record (patients with 0 assessments are not included). rw-response, real-world response.

Overall, the availability of images was variable and low across cohorts, indicating the need to rely on other data components to assess rw-response (Fig 3A, Data Supplement, Table S10).

#### Images

Four of seven cohorts had images available for a subset of patients, with 35% (mean range, 1.5%-98%) of patients with images available across cohorts. Although images were available for four cohorts, only two had extractable images (ie, diagnostic-quality radiologic images accessible to the data provider for reading by an independent radiologist; Data Supplement, Table S10). For those patients with at least one image available, there was a median of three images per patient (range, 1-6; Fig 3B, Data Supplement, Table S10).

#### Image Reports

Almost all cohorts (6/7) had at least one image report available per patient for the majority of patients (median 97% of patients, range, 67.5%-100%; Fig 3A, Data Supplement, Table S11). For those patients with at least one image report available, there was a median of four image reports (range, 3-6) per patient (Fig 3B, Data Supplement, Table S11). The proportion of patients with both baseline and postbaseline image reports was lower, with a median 75% of patients with both (range, 55%-85.6%; Table 1). The timing of both imaging and image reports from baseline to postbaseline and on treatment varied across cohorts (Table 1, Data Supplement, Table S12). Additionally, multiple imaging modalities were used for most patients (Data Supplement, Fig S1), with CT being the most frequent. The indication for imaging, as determined from the image report, was most frequently described as for initial baseline or scheduled surveillance, rather than for clinically suspected recurrence (Data Supplement, Fig S2).

**TABLE 1.** Medians Across Cohorts Calculated From Summary-Level Statistics of Each Cohort

Component	Baseline to Index	Baseline to Postbaseline	First to Second Postbaseline
Images			
Proportion with data, median (range)	28% (1.5%-92%)	22% (0.5%-79.5%)	29% (0.5%-86%)
Time between in weeks, median (range)	2.95 (2.4-5)	13.2 (7.3-18)	6 (3.29-7)
Image reports			
Proportion with data, median (range)	88.80% (63.5%-98.3%)	75% (55%-85.6%)	85% (59%-87.2%)
Time between in weeks, median (range)	3.63 (2.3-4)	9.62 (7.5-18)	5 (3.7-6.3)

	Index to Assessment	First to Second Assessment
Clinician assessment		
Proportion with data, median (range)	77.50% (74%-88.3%)	44.50% (32%-61%)
Time between in weeks, median (range)	7.9 (6.9-8)	7.9 (6-9)

NOTE. The median time between data components is calculated only for patients with at least one data component in the record (patients with 0 assessments are not included).

#### Clinician Assessment:

All data sets had clinician assessments available for most patients (median 77.5% of patients, range, 74%-88.3%; Fig 3A). For those patients with a clinician assessment available, a median of two assessments per patient was observed for 6/7 cohorts and one cohort with a median of one assessment per patient (Fig 3B). A median of 44.5% patients across cohorts (range, 32%-61%) had more than one clinician assessment (Data Supplement, Table S13). The timing of clinician assessments was relatively consistent across cohorts, with a median of 7.9 weeks between both the index date to first assessment and first to second assessment (Table 1, Data Supplement, Table S13). Across all cohorts, imaging was the most frequently cited source of evidence for clinician assessments of response (Data Supplement, Fig S3), followed by symptoms.

### rw-Response Estimates and End Points

**FIG 4.**
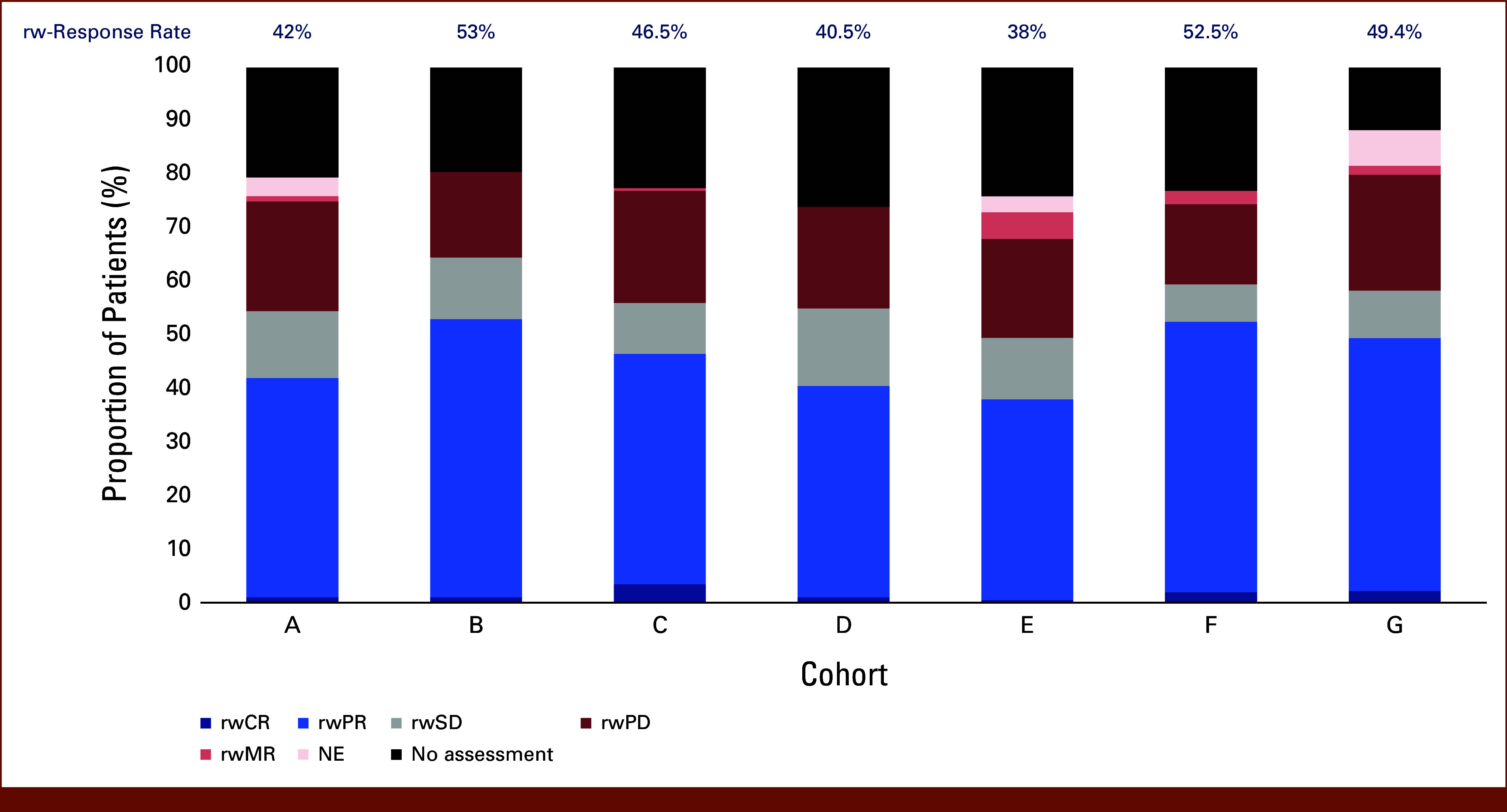
rw-best overall response and response rate across cohorts. The proportion of patients in each cohort with a given rw-best overall response by clinician assessment of response. Response rate (above bars) is derived from patients with rwPR and rwCR, out of total patients. Cohorts A-F, n = 200 patients; cohort G, n = 180 patients. NE, not evaluable; rw, real-world; rw-response, real-world response; rwCR, rw-complete response; rwMR, rw-mixed response; rwPD, rw-progressive disease; rwPR, rw-partial response; rwSD, rw-stable disease.

**FIG 5.**
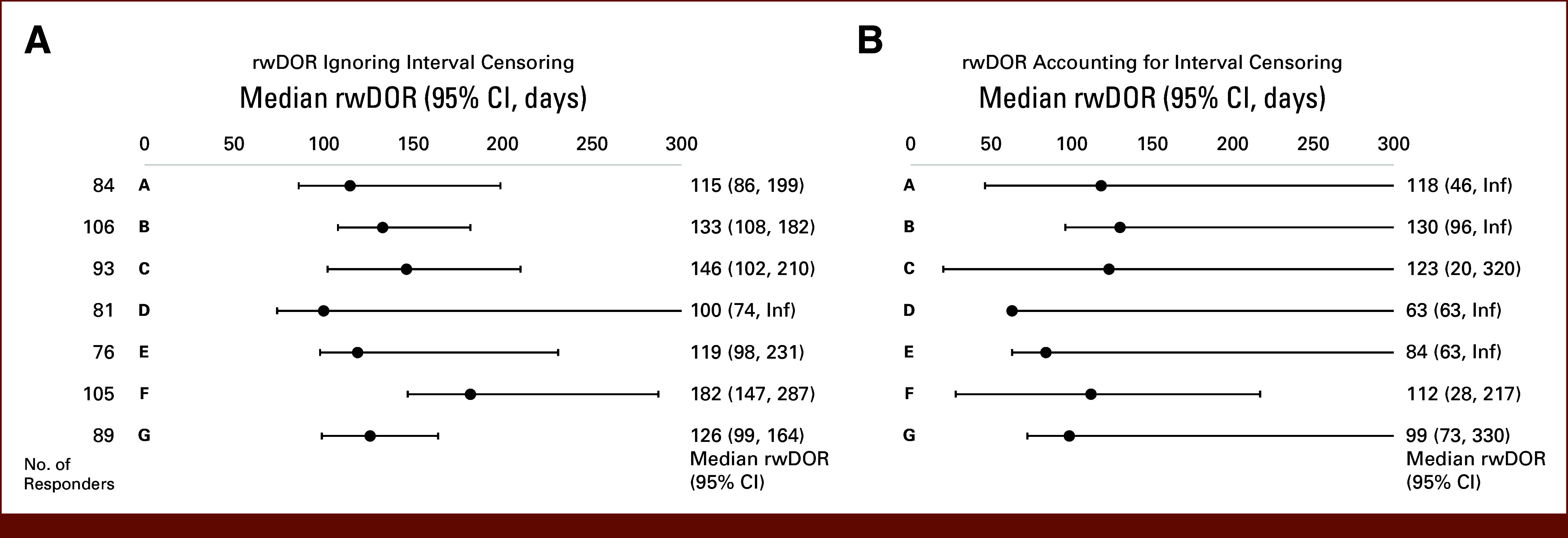
rwDOR across cohorts. rwDOR (A) ignoring interval censoring and (B) accounting for interval censoring for patients with complete or partial response (responders) across cohorts. Graphs show the median rwDOR with 95% CIs. rwDOR, real-world duration of response.

There was relative consistency in rwRR (median, 46.5%, range, 38%-53%) using clinician-documented response across cohorts (Fig 4). A median of 22.5% (range, 11.7%-26.0%) of patients did not have a response assessment during the assessment period, and these patients had the shortest follow-up time compared with responders and nonresponders (Data Supplement, Fig S4). There was variability in rwDOR across data sets (Fig 5, Data Supplement, Fig S5), and accounting for interval censoring substantially increased the estimated variance.

The results of the sensitivity analyses were relatively consistent with the primary analyses (Data Supplement, Table S14).

**FIG 6.**
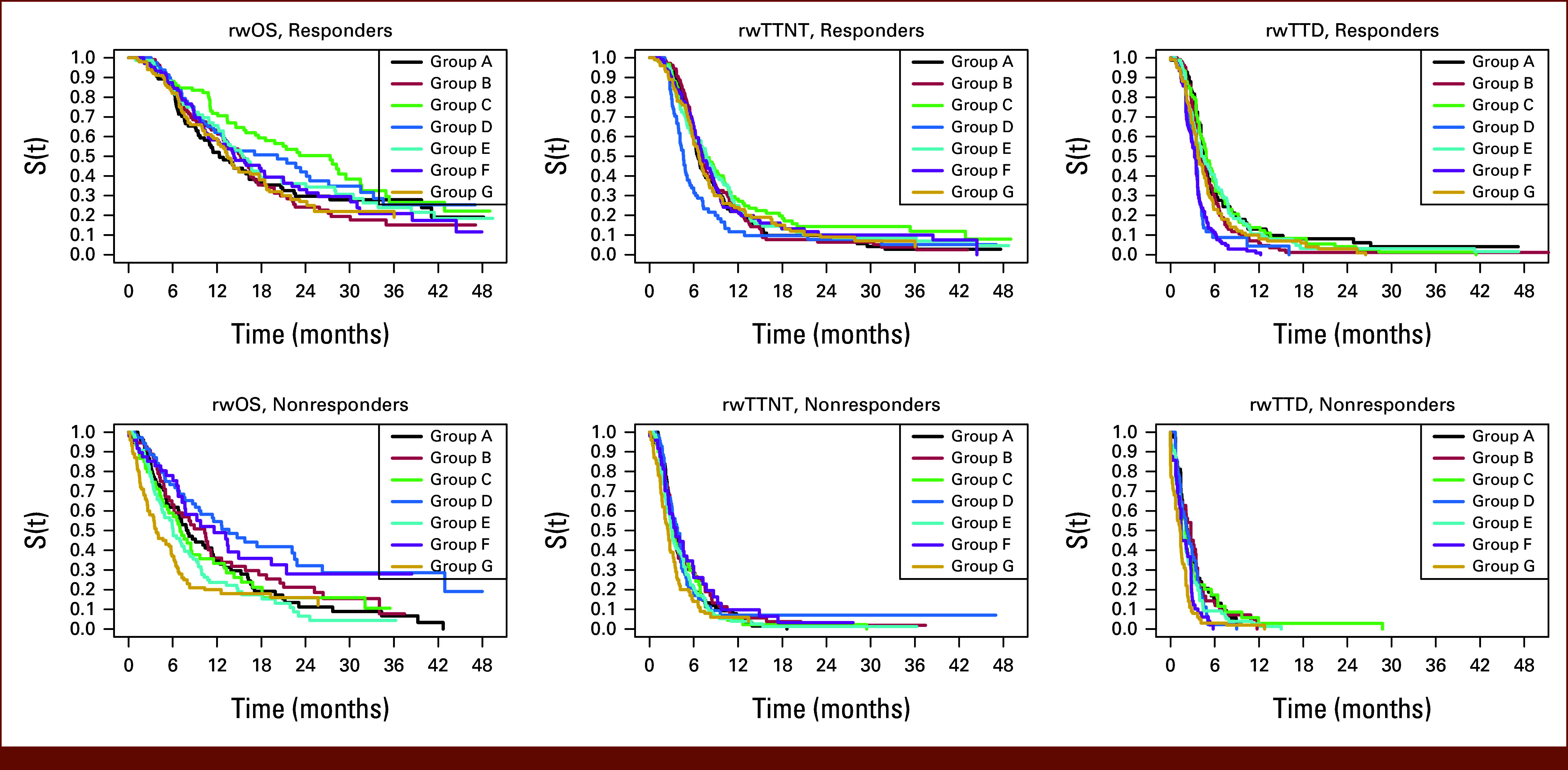
rw-time to event end points by rw-response to treatment. Kaplan‐Meier curves for responders and nonresponders for rwOS, rwTTNT, and rwTTD, across cohorts. rw, real-world; rwOS, rw-overall survival; rwTTD, rw-time to treatment discontinuation; rwTTNT, rw-time to next treatment.

The relationships between rw-response and rwOS, rwTTD, and rwTTNT were analyzed. Relative consistency was observed in the median estimates and directionality of the time-to-event end points (rwOS, rwTTD, and rwTTNT) across cohorts for responders compared with nonresponders (Fig 6, Data Supplement, Fig S6). Like the short follow-up time seen for patients with no response assessment, rwTTD, rwTTNT, and rwOS were consistently shorter for those with no response assessment than for both nonresponders and responders (Data Supplement, Fig S6).

## DISCUSSION

Overall, this collaborative effort assessed the availability of data components to measure rw-response and evaluated the consistency of the measure across RWD sources. The pilot demonstrates the feasibility of aggregating data from various rw-data sets to generate RWE. Findings highlight reasonable consistency in rw-response across disparate data sources in an aligned patient population using clinician-documented response.

The pilot used a common protocol, with all data providers following an a priori agreed upon eligibility criteria, statistical analysis plans, and standardized definitions. For this methodological exercise, the patient population reflected previous *Friends*' pilots during a time frame when chemotherapy was frequently used, focusing on PDC to remove potential confounding of pseudoprogression with immunotherapy treatment.

Using RWD for causal inference can be challenging for many reasons, including the need to ascertain relevant and reliably detailed, longitudinal clinical characteristics. Data generation currently requires significant manual abstraction and curation, which limited the sample size, highlighting the challenges with evaluating rw-response and the need for standardized structured RWD. RWD can be generated from multiple sources, including EHR-derived and administrative claims data; however, EHR data were necessary to ascertain rw-response. Although many areas showed relative consistency across EHR-derived RWD cohorts, areas such as specific clinical characteristics (eg, other treatment modalities) and availability of imaging were more variable or limited for some cohorts. To support causal inference, other variables must be appropriately controlled to demonstrate that tumor response is due to the treatment, not factors such as concomitant therapies, additional modalities, or other confounding factors.

The availability and extractability of images was limited and varied significantly across cohorts. Privacy, contractual, and/or compliance issues were stated as barriers to obtaining and sharing images. Additionally, linking images to the EHR requires a high level of interoperability, data management (privacy and deidentification considerations), and storage that may not be feasible for all institutions. This remains a technological and infrastructural challenge to using rw-end points.

Ascertaining rw-response from currently available EHR data will likely need to rely on clinician assessments. Response evaluated by the clinician's assessment of a patient's change in disease burden was available for most patients across all cohorts. Multiple imaging modalities were used, which may make applying a RECIST-like assessment of response difficult. The clinician assessment considers a variety of inputs (eg, radiology, physical examination, biomarkers, pathology, and patient-reported symptoms), which introduces heterogeneity and subjectivity, although findings reported herein demonstrate the source of evidence for most assessments was imaging and image reports. The timing of clinician assessments was relatively consistent across cohorts and reflects the timing prescribed in PDC clinical trials where patients are assessed every 6-8 weeks after random assignment, indicating that patients treated outside clinical trials are likely under similar active assessment or surveillance at regular intervals. However, a proportion of patients did not have a response assessment, possibly due to being lost to follow-up, rapid decline, transfer of patient care, discontinuation of treatment because of toxicity, or patient choice.

Using clinician assessment to evaluate rwRR was relatively consistent across all RWD sources, albeit notably higher than values observed in mNSCLC trials for patients treated with PDC (rwRR median 46.5% compared with ORRs of 19.4%^11^ and 38.4%^12^). Given the lack of application of standardized RECIST assessment criteria outside of clinical trials, a rwPR can include any reduction of the tumor burden, not the minimum of 30% reduction required by RECIST 1.1. Likewise, the results showed a median of 11.5% of patients classified as rwBOR of rwSD, while the trials referenced above had 51% and 37% of patients classified as having stable disease, respectively. Therefore, patients with small decreases in tumor burden in routine clinical practice may be categorized as partial responders, while these same patients would likely be categorized as stable disease based on RECIST 1.1 criteria. Durability of response can provide additional insight into therapeutic efficacy, and rwDOR varied across cohorts in the study, possibly because of the variability in timing of patient assessments, variability in reporting of data, or other unmeasured or residual factors.

This study has several limitations. Data were aggregated from various data providers, such that duplication of patients may have occurred, and therefore data in the different cohorts may contain some of the same patients. Furthermore, interval censoring may have made interpretation challenging. The study also did not require patients to have measurable disease, as would be required in clinical trials using RECIST. Finally, although each data provider used patient-level data, aggregate analyses across cohorts were limited to interpretations from summary-level data.

The demonstrated feasibility of data providers' adherence to a common data model with relative consistency in rw-response end points on the basis of clinician assessment suggests rw-response warrants further exploration to inform drug effectiveness evaluation. There is a degree of uncertainty in the relationship between RECIST-based assessment and clinician assessment, which requires additional methodological development. Therefore, rw-response end points are not directly comparable with RECIST-based clinical trial response assessments and may best be leveraged for evaluation of response within RWD. Use of rw-response may support evaluation of a treatment effect in a specific population in the rw-setting or in subpopulations that were underrepresented in clinical trials. The measure may also be valuable for signal seeking to aid in identifying populations in which to explore efficacy in future clinical trials or for evidence to support label expansion of an already approved therapy. Aligning methodologies for aggregating and analyzing RWD will support use of RWD as a reliable and consistent source of RWE to support oncology drug development and regulatory decision making.
